# Validation of the Turkey Semen Cryopreservation by Evaluating the Effect of Two Diluents and the Inseminating Doses

**DOI:** 10.3390/ani10081329

**Published:** 2020-08-01

**Authors:** Michele Di Iorio, Giusy Rusco, Roberta Iampietro, Lucia Maiuro, Achille Schiavone, Silvia Cerolini, Nicolaia Iaffaldano

**Affiliations:** 1 Department of Agricultural, Environmental and Food Sciences, University of Molise, 86100 Campobasso CB, Italy; giusyrusco@gmail.com (G.R.); robertaiampietro@hotmail.it (R.I.); maiuro@unimol.it (L.M.); nicolaia@unimol.it (N.I.); 2 Department of Veterinary Science, University of Turin, 10095 Torino TO, Italy; achille.schiavone@unito.it; 3 Department of Veterinary Medicine, University of Milan, 20122 Milano MI, Italy; silvia.cerolini@unimi.it

**Keywords:** turkey semen, extender, inseminating dose, fertility, hatching rate

## Abstract

**Simple Summary:**

Achieving an effective freezing protocol, that is able to preserve the fertilizing ability of turkey semen, is a key aim for the establishment of the first national semen cryobank of autochthonous chicken and turkey breeds within our national project (Tutela della biodiversità nelle razze avicole italiane—TuBAvI). In this regard, we have performed different studies in order to define the best conditions for cryopreservation of turkey semen; namely, we identified an effective freezing protocol which is based on the use of dimethylsulfoxide as a permeant cryoprotectant (CPA) combined with Ficoll as a non-permeant CPA. Here, our purpose was to test this protocol in vivo, by evaluating the effect of two extenders and three inseminating doses. The good fertility and hatching rates achieved here are promising for future studies, in which our cryopreservation protocol will be tested on Italian autochthonous turkey breeds and also to the advantages offered by the extensive use of frozen semen in the turkey breeding industry.

**Abstract:**

This study was designed to test the fertilizing ability of cryopreserved turkey semen, and here, two experiments were performed: an in vitro analysis to assess the effects of Tselutin and Lake diluents and an in vivo test to determine the fertility and hatching rates by also studying the feat of three insemination doses (250, 400 and 600 × 10^6^ sperm/hen). Pooled semen samples were diluted with Tselutin or Lake extender which contained 20% of dimethylsulfoxide and 1 mM of Ficoll at final sperm concentration of 3 × 10^9^ sperm/mL. Thereafter, semen was packaged into straws and frozen on liquid nitrogen. The post-thaw sperm quality was evaluated considering motility (computer-aided sperm analysis—CASA system) and membrane integrity (flow cytometry). Significantly higher values of progressive motility and some kinetic parameters in semen frozen with Lake were found. When we compared the extenders in vivo, no significant effects were detected, whilst sperm concentration significantly affected both fertility and hatching rates, with the best results obtained with the sperm concentration of 400 × 10^6^ sperm/hen. From the results obtained, it emerged that the extender type only affected sperm motility characteristics, not the fertilizing ability of frozen-thawed semen, while inseminating dose markedly affected fertility and hatching rates.

## 1. Introduction

The semen cryopreservation in avian species is the safest and most reliable strategy for the in vitro conservation of genetic resources and safeguarding of rare breeds [1,2,3,4]. The development of an effective semen freezing protocol in avian species represents one of the most important challenges for the conservation of genetic variability, through the implementation of a semen cryobank [1,2,4,5,6,7,8].

In this regard, thanks to the financed project “Tutela della biodiversità nelle razze avicole italiane—TuBAvI” by the Ministry of Agricultural, Food and Forestry Policies (MiPAAF), some Italian research groups are engaged in the identification of an effective semen freezing protocol for avian species (*Gallus gallus* and *Meleagris gallopavo)* in order to create the first semen cryobank of autochthonous chicken and turkey breeds in Italy.

In *Meleagris gallopavo*, semen cryopreservation, beyond guaranteeing the conservation of genetic resources in a gene bank, could have important practical advantages for artificial insemination (AI) in intensive breeding. In this regard, the mating of turkeys on commercial farms is completely dependent on AI to obtain fertile eggs, because their oversized and heavy breasts of males make it impossible to mate naturally.

The cryopreservation process causes numerous negative effects including damages to cell membranes (plasma and mitochondrial) and, in some cases, to the nucleus, and that has devastating consequences for sperm survival and function [7]. It is widely recognized that the ability of avian spermatozoa to survive and remain functional in sperm storage tubules is significantly compromised after the freezing/thawing process [5,6,9,10]. This has an inevitable negative impact on the fertilizing capacity when cryopreserved sperm is used [7,8,11,12,13].

Thus, the conservation of the sperm structure and its functionality strictly depends on the cryopreservation protocol used [8].

During the last few decades, several studies have been conducted to find an effective freezing procedure for turkey semen, in which different variables involved in sperm cryosurvivability were taken into consideration: extender, dilution rate, cryoprotectant (CPA), freezing conditions, packaging system and warming procedure [6,7,12,14,15,16,17]. According to the literature, a wide variability of results of both the frozen turkey semen quality (motility ranging from 10–60% and the viability ranging from 15–84%) [6,7,15,16,17,18,19,20,21] and its fertilizing capacity (ranging from 0–84%) [6,22,23] were reported.

In the last few years, we have identified in vitro as an effective freezing protocol which is based on the use of dimethylsulfoxide (DMSO) as a permeant cryoprotectant (P-CPA) combined with Ficoll as a non-permeant cryoprotectant (NP-CPA) [7,24], with a final sperm concentration of 3 × 10^9^ sperm/mL.

However, despite our efforts and encouraging results obtained so far, we still aim to obtain a freezing procedure with a fertilization rate as similar as possible to that of fresh semen.

Some of the parameters that we have not yet evaluated in the freezing protocol are the assessment of other based extenders and the appropriate insemination dose that will allow us to write up national semen cryobank guidelines. In addition, these findings could improve the current prospects for the commercial use of frozen turkey semen.

Thus, we are going to compare our standard extender (Tselutin) to the Lake extender on in vitro and in vivo cryosurvival of turkey semen. Recently, the Lake extender has been used successfully for cryopreservation of chicken spermatozoa [4,25] with excellent results in vivo [4]. In such context, the goal of this paper was to compare the effects of Tselutin vs. Lake: (1) in vitro, by assessing the post-thawed sperm motility and viability, and (2) in vivo, by evaluating the fertilizing ability of cryopreserved semen also using three inseminating doses.

## 2. Materials and Methods

### 2.1. Chemicals

All chemicals used were of the highest commercially available purity. Unless stated otherwise, all of the chemicals were purchased from Sigma, Chemical Co. (Milan, Italy).

### 2.2. Animals

The animals used during this study were Hybrid Large White line British United Turkeys (B.U.T.). Forty turkey males and 126 turkey hens that were supplied by Agricola Santo Stefano (Amadori Group, TE, Italy). Turkeys were reared in a poultry house with a controlled environment that had artificial lighting (14 h light–10 h dark cycle) and all animals were given free access to a standard commercial feed and water.

The experiments were carried out in accordance with the Code of Ethics of the EU Directive 2010/63/EU for animal experiments. The approval request number was 2020-UNMLCLE-20232.

However, all procedures reported in this work and that contribute to the care and use, including the semen collection of Hybrid Large White turkey male (Aviagen turkey, 31186 Midland Trail, East Lewisburg, WV 24901, USA), were performed at a commercial Amadori breeding center that complies with the ethical standards of the Aviagen guides. No animal was anaesthetized, mistreated or sacrificed during this study. Semen samples were routinely collected as part of the standard management procedure for male turkey breeders at the breeding farm.

### 2.3. Experiment 1: Effects of Lake and Tselutin Extender on In Vitro Post-Thaw Quality of Turkey Semen

#### 2.3.1. Semen Collection and Processing

This experiment was conducted during the period of May–July 2019, and the toms were 32 weeks old at the beginning of the experiment.

This period overlapped with the height of the reproductive period of the toms (32nd–44th week), which made it the best period to obtain semen with good quality [26,27].

Semen was obtained from toms through abdominal massage and pooled (1 ejaculate/male; 5 ejaculates/pool). Six pools were used, and the quality of each pool was evaluated immediately after collection as described below (see sperm quality). Subsequently, each pool was divided into two equal aliquots, diluted with Tselutin or Lake extender (Table 1), in order to obtain a sperm concentration of 6 × 10^9^ spermatozoa/mL, which was cooled at 4 °C for 25 min. Thereafter, the pre-extended semen was further diluted (1:1, v/v) with the freezing medium composed of Lake or Tselutin extender, both containing 20% of dimethylsulfoxide (DMSO) as P-CPA and 1 mM of Ficoll 70 as NP-CPA [24] to reach a final sperm concentration of 3 × 10^9^ spermatozoa/mL. The diluted semen was loaded into 0.25 mL straws through the aid of a manual micro-aspirator (IMV-Technologies, Piacenza, Italy) and then equilibrated at 4 °C for 20 min. Straws were frozen and thawed in accordance to our previous studies [7,24].

#### 2.3.2. Sperm Quality

Sperm motility characteristics and viability were both assessed in duplicate in fresh and thawed semen samples. Sperm motility was estimated using a computer-aided sperm analysis system coupled with a phase contrast microscope (Nikon Eclipse model 50i; negative contrast, Firenze, Italy) using Sperm Class Analyzer (SCA) software (version 4.0, Microptic S.L., Barcelona, Spain). The samples were diluted to 0.9% NaCl to reach a final sperm concentration of 100 × 10^6^/mL. After an incubation of 5 min at 38 °C, a semen aliquot (5 µL) was allocated onto a microscope slide and observed under the microscope at 100× total magnification. The following parameters were recorded: total motility (%), progressive motility (%), curvilinear velocity (VCL, (µm/s)), straight-line velocity (VSL, (µm/s)), average path velocity (VAP, (µm/s)), linearity (LIN, (%)) and straightness (STR, (%)). At least 500 sperm for each sample were observed.

Sperm viability was measured using the Muse^®^ Cell Analyzer (Luminex corporation, 12212 Technology Blvd Suite 130, Austin, TX 78727, United States) following the manufacturer’s protocol. Semen samples were diluted in Phosphate-buffered saline (PBS) so that a concentration ranging from 1 × 10^5^ to 1 × 10^7^ spermatozoa/mL could be reached. Subsequently, 20 μL of this suspension was mixed with 780 μL (dilution factor 1:40) of a Muse Count and Viability Kit^®^ in an Eppendorf tube (Luminex corporation) and incubated for 5 min at room temperature. Subsequently, the Eppendorf were analyzed with the flow cytometry. Then, the software module performed calculations and displayed data in two dot plots: (1) nucleated cells: a membrane-permeant DNA staining dye that stained all cells with a nucleus. This plot functions to identify cells with a nucleus from debris and non-nucleated cells. (2) Viability: a DNA-binding dye stains cells that had lost their membrane integrity and allows the dye to stain the nucleus of dead and dying cells. This parameter discriminates viable (live cells that do not stain) from non-viable (dead or dying cells that stain).

### 2.4. Experiment 2: Effect of Extender and Inseminating Dose on the Reproductive Performance of Turkey Hens

In this experiment, we tested the fertilizing ability of three sperm concentrations per insemination dose in the presence of Lake or Tselutin diluent. The 126 turkey hens were divided into seven treatment groups (18 hens in each group) and inseminated with the volume and sperm concentration as reported in Table 2. The hens of each control group were inseminated with fresh semen abiding the standard procedures of the breeding which had the dilution 1:10 with a commercial extender and inseminating dose per hen of 50 µL.

In particular, over the period of 2 weeks, two intravaginal artificial inseminations were performed, one on 23 August and another on 30 August, using fresh or frozen-thawed semen, respectively.

At the moment of insemination, the cryopreserved semen was thawed at 50 °C for 10 s.

Egg collection began after the second insemination and went on for 8 days, for each group 102 ± 11 egg were gathered.

The eggs were incubated at 37.8 °C and a relative humidity of around 60% at the Amadori group (Bertinoro, Forlì-Cesena, Italy) hatchery. Eggs were candled on the 15th day, unfertilized eggs and eggs with dead embryo were discarded.

Fertility and hatching rates were calculated using the following formulas:

(1) $$Fertility\;rate=\frac{n{}^{\circ}\;fertile\;eggs}{total\;n{}^{\circ}\;incubated\;eggs}\times 100$$

(2) $$Hatching\;rate=\frac{n{}^{\circ}\;hatched\;eggs}{total\;n{}^{\circ}\;incubated\;eggs}\times 100$$

### 2.5. Statistical Analysis

The effect of the extenders on in vitro sperm variables (computer-aided sperm analysis—CASA motility parameters and viability) was tested by independent-sample *t*-test. Fertility and hatching rates were compared among fresh semen and semen frozen in the presence of different extenders and inseminating doses by analysis of variance (ANOVA), followed by Duncan’s comparison test.

To compare the different treatments in vivo, we used a generalized linear model (GLM) procedure to determine the fixed effect of the extender, insemination doses and their interaction for the sperm fertility and hatching rates. These last parameters were measured across the treatments of frozen semen (2 extenders × 3 sperm concentration) and were compared by analysis of variance (ANOVA) followed by Duncan’s comparison test. Significance was set at *p* < 0.05 and every statistical test was performed using the software package SPSS (SPSS 15.0 for Windows, 2006; SPSS, Chicago, IL, USA).

## 3. Results

### 3.1. Fresh Semen Quality

The semen quality parameters recorded from the pools of freshly collected semen are reported in Table 3. The average sperm concentration was about 9 × 10^9^ sperm/mL. Total sperm motility was higher than 80% and progressive motility was 26%. Sperm viability was recorded in more than 90% of the sperm population. These features indicate an adequate fresh sperm quality which represents an important requirement for its utilization in the cryopreservation process.

### 3.2. Effect of Extender on In Vitro Post-Thaw Semen Quality

The sperm quality post-thawing resulted was worse with respect to fresh sperm. In particular, we observed a loss of about 55 to 60% for total motility, 85% for progressive motility, 40 to 60% for related kinetic parameters and 45% for sperm viability.

Sperm motility parameters and sperm viability obtained in semen frozen in the presence of Lake and Tselutin diluent are provided in Table 4. Significantly higher values of progressive motility, VCL, VAP, LIN, wobble (WOB) and amplitude of lateral head displacement (ALH) were found in semen diluted and cryopreserved with Lake extender compared to Tselutin. No significant differences were detected for sperm viability, total motility, VSL, STR and BCF.

Table 5 provides the percentage of fertility and hatching rates obtained after intravaginal artificial insemination of turkey hens with fresh and frozen semen.

No significant effect of extender was detected for fertility and hatching rates, whilst sperm concentration significantly affected both parameters evaluated. Moreover, the interaction between extender and sperm concentration had no significant effect on the variables examined.

Significantly higher values of fertility and hatching rates were recorded in fresh semen with respect to that with frozen treatment, except for those with Lake/400 ×10^6^ for fertility and Tselutin/250 × 10^6^ and 400 × 10^6^ for hatching rate. However, the treatment with Lake and insemination dose of 400 × 10^6^ spermatozoa (spz), guaranteed significantly higher values of fertility compared to all other frozen semen treatments. Higher hatching rates were recorded with the treatments of Tselutin/250 × 10^6^.

## 4. Discussion

In the present study, in order to obtain a freezing procedure with a fertilization rate as similar as possible to that of fresh semen, we investigated the effect of the two extenders (Tselutin and Lake) on in vitro sperm cryosurvival and on the fertilizing ability by testing three sperm concentration doses. It is to be highlighted that for the first time, we evaluated the fertilizing ability by testing three sperm concentration doses using the optimized freezing protocol [24]. Examining the results obtained in vitro it emerged that the Lake extender preserved the post-thaw progressive sperm motility and related kinetic parameters better (*p* < 0.05). This could be attributed to its chemical composition; in this regard, we speculate that the Lake extender would provide more appropriate nutrients for the semen cryopreservation as an energy source, chemical compounds that buffer against harmful changes of pH and provide a physiological osmotic pressure and concentration of electrolytes.

From the investigation of the composition of two extenders, it emerged that the Lake extender contained fructose, whilst the Tselutin one contained glucose. Previous research showed that turkey spermatozoa utilize fructose more efficiently than glucose [28,29].

In addition, Lake includes the polyvinylpyrrolidone (PVP), whereas this is absent in Tselutin. The PVP is a water-soluble polymer, which is identified as a NP-CPA at a high molecular weight by various authors [4,13,30,31,32]. Thus, we can speculate that there is a synergistic effect between PVP and Ficoll that offers more effective dehydration to the sperm cells during the freezing process. This prompts a reduction of ice crystal formation, which is the main biophysical mechanism causing sperm death that happens during the cryopreservation process.

Regarding in vivo parameters, no significant effect of extender was found, whilst the sperm concentration significantly affected the fertilization and hatching rates. Excellent values of fertility and hatching rates, up to 87.2%, using the concentration of 400 × 10^6^ sperm/hen for both extenders tested were obtained. In addition, no interaction between extender × sperm concentration was established.

In accordance with standard procedure of the Amadori farm, we used the fresh semen doses of 0.05 mL with a sperm concentration of about 250 × 10^6^ derived from a semen dilution of 1:10. This sperm concentration is consistent with previous authors, who reported that the optimal fresh sperm concentration per inseminating dose was 200–250 × 10^6^ sperm [33,34,35,36]. Thus, in order to compensate for the lower freezing sperm quality that was registered, we also tested concentrations higher than 250 × 10^6^, i.e., semen doses of 400 and 600 × 10^6^ sperm/per hen. The semen concentration of 250 × 10^6^/hen was insufficient to produce an appropriate reproductive performance, whilst that of 400 × 10^6^ both in the presence of Lake and Tselutin generated 87.2% and 80.9% fertility, and 70.9% and 68.5% hatching rates respectively.

Surprisingly, we did not observe an improvement of fertility and hatching rates with the higher semen dose (600 × 10^6^); on the contrary, a significant decrease was observed, particularly for Lake diluent. This could be due to the fact that 600 ×10^6^/hen also includes a higher volume of semen used to inseminate with respect to the other inseminating doses. Thus, we hypothesize that a semen reflux can occur with a consequent reduction in the number of spermatozoa able to reach the sperm storage tubules (SST).

Here, the fertility and hatching rates achieved were at satisfactory levels considering some discouraging and variable results reported in the literature about reproductive performances when cryopreserved semen is used. The diverse outcomes are due to variability in the biological material and the multiplicity of preservation procedures used. In this regard, Tselutin et al. [22] obtained a fertility rate ranging from 71−84.3% using the pellet method as a freezing packaging system, Labbé et al. [23] recorded values of fertility ranging from 20% using straws and up to 38% with the pellet method and Long et al. [6] registered the value of 32.6% as the highest fertility when cryopreserved semen in straws were used.

In addition, these latter authors [6] also suggested to use repeated inseminations 2 or 3 times per week in order to compensate for the lower concentrations of motile and functionally competent sperm in frozen semen compared to fresh semen.

In this research, the reproductive performance might be overestimated because our fertility trial was performed on hens from commercial breeding at the end of their reproductive cycle. The hens had been inseminated with fresh semen weekly during the reproductive cycle in order to maintain an adequate reserve of sperm in sperm storage tubules (SST), and thus ensure high fertility rates. Hence, the spermatozoa from fresh semen doses could have remained in the hen oviduct for a long time and affected the reproductive performances obtained with frozen semen. Spermatozoa can survive up to 10 weeks in SST of the turkey hens [37,38,39,40], although the fertility rates drop progressively each week [6].

In light of this, we are hoping to back up these results using hens that have not been previously inseminated with fresh semen.

## 5. Conclusions

In conclusion, from the results obtained, it emerged that the extender type only affected sperm motility characteristics, not the fertilizing ability of frozen-thawed semen, while inseminating dose markedly affected fertility and hatching rates.

Finding an efficient freezing protocol for turkey semen and determining the appropriate inseminating dose and frequency is vital for the establishment of the first national semen cryobank within our national project (TuBAvI).

In this regard, the fertility and hatching rates achieved here are promising for future studies that will aim to test the semen cryopreservation on Italian autochthonous turkey breeds and also to improve current prospects for the commercial use of frozen turkey semen.

## Figures and Tables

**Table 1:** Chemical composition of Tselutin and Lake extender.

Components	Tselutin mM	Laken mM
Glucose	44.4	-
Fructose	-	44.4
Sodium glutamate	128.0	102.6
Di-Potassium hydrogen phosphate	20.0	-
Potassium acetate	-	50.9
Magnesium acetate	7.0	4.91
Glycine	13.3	-
Glutamic acid	7.68	-
Inositol	11.1	-
Polyvinylpyrrolidone	-	0.3
pH	6.65	7.00

**Table 2:** Experimental groups of turkey hens used in the artificial insemination trial.

	Group	Hens Number	Extender	Semen Volume µL	Sperm Concentration × 10^6^
Fresh	1	18		50	250
Frozen	2	18	Lake	200	600
	3	18	Tselutin	200	600
	4	18	Lake	135	400
	5	18	Tselutin	135	400
	6	18	Lake	85	250
	7	18	Tselutin	85	250

**Table 3:** Sperm quality variables (means ± standard error of means—SEM) recorded in freshly collected turkey semen (n = 6).

Sperm Variables	Mean ± SEM
Total motility (%)	82.2 ± 1.2
Progressive motility (%)	26.2 ± 2.2
VCL (µm/sec)	60.1 ± 3.9
VAP (µm/sec)	41.4 ± 3.6
VSL (µm/sec)	27.8 ± 2.2
STR (%)	56.1 ± 3.5
LIN (%)	35.1 ± 2.4
WOB (%)	55.3 ± 2.4
ALH (µm)	2.8 ± 0.2
BCF (Hz)	4.6 ± 0.4
Viability (%)	91.8 ± 0.8
Concentration (× 10^9^/mL)	9.1 ± 0.5

VCL: curvilinear velocity; VAP: average path velocity; VSL: straight-line velocity; STR: straightness; LIN: linearity; WOB: wobble; ALH: amplitude of lateral head displacement; BCF: beat cross frequency.

**Table 4:** Probability of *t*-test and means ±SEM of sperm quality parameters recorded in thawed turkey semen samples (n = 6) frozen in the presence of Lake and Tselutin extender.

Sperm Parameters	Lake	Tselutin	*p*-Value
Total motility (%)	35.8 ± 2.2	31.4 ± 1.0	0.105
Progressive motility (%)	4.1 ± 0.6 ^a^	2.5 ± 0.3 ^b^	0.031
VCL (µm/sec)	36.1 ± 1.6 ^a^	30.2 ± 1.7 ^b^	0.032
VAP (µm/sec)	16.9 ± 0.9 ^a^	13.1 ± 1.1 ^b^	0.022
VSL (µm/sec)	9.3 ± 0.8	7.0 ± 0.8	0.072
STR (%)	43.4 ± 1.0	39.9 ± 1.2	0.053
LIN (%)	22.1 ± 1.5 ^a^	17.3 ± 1.4 ^b^	0.041
WOB (%)	42.8 ± 1.6 ^a^	36.7 ± 1.6 ^b^	0.024
ALH (µm)	2.1 ± 0.1 ^a^	1.8 ± 0.1 ^b^	0.016
BCF (Hz)	2.4 ± 0.2	1.9 ± 0.1	0.067
Viability (%)	47.4 ± 1.5	50.9 ± 1.7	0.156

^a,b^ Means ± standard error of means (SEM) within the same row differ significantly at *p* < 0.05 according to Student’s *t*-test procedure. VCL: curvilinear velocity; VAP: average path velocity; VSL: straight-line velocity; STR: straightness; LIN: linearity; WOB: wobble; ALH: amplitude of lateral head displacement; BCF: beat cross frequency.

**Table 5:** Fertility and hatching rates in turkey hens fertilized with fresh and frozen semen in the presence of Lake or Tselutin diluent and three sperm concentrations per insemination dose.

Semen Treatment	Extender	Sperm Concentration (× 10^6^ spz)	Total Fertile Eggs (%)	Hatching Rate (%)
Fresh	-	250	90.8 ^a^	75.6 ^a^
Frozen	Tselutin	600	81.9 ^bc^	51.4 ^d^
		400	80.9 ^bc^	68.5 ^abc^
		250	82.2 ^bc^	72.3 ^ab^
	Lake	600	74.3 ^c^	58.4 ^cd^
		400	87.2 ^ab^	70.9 ^ab^
		250	80.7 ^bc^	62.5 ^bcd^
Extender effect			*p* = 0.767	*p* = 0.974
Sperm concentration effect			*p* = 0.049	*p* = 0.004
Extender × sperm concentration effect			*p* = 0.193	*p* = 0.201

^a–d^ Values within a column reporting different a superscript letter differs significantly at *p* < 0.05. spz: spermatozoa.
